# Polyphenolic Extract from Tarocco (*Citrus sinensis* L. Osbeck) Clone “Lempso” Exerts Anti-Inflammatory and Antioxidant Effects via NF-kB and Nrf-2 Activation in Murine Macrophages

**DOI:** 10.3390/nu10121961

**Published:** 2018-12-11

**Authors:** Giacomo Pepe, Eduardo Sommella, Donato Cianciarulo, Carmine Ostacolo, Michele Manfra, Veronica Di Sarno, Simona Musella, Mariateresa Russo, Antonella Messore, Barbara Parrino, Alessia Bertamino, Giuseppina Autore, Stefania Marzocco, Pietro Campiglia

**Affiliations:** 1 Department of Pharmacy, School of Pharmacy, University of Salerno, Via Giovanni Paolo II 132, I-84084 Fisciano, Italy; gipepe@unisa.it (G.P.); esommella@unisa.it (E.S.); donatocianciarulo@hotmail.com (D.C.); vdisarno@unisa.it (V.D.S.); smusella@unisa.it (S.M.); abertamino@unisa.it (A.B.); autore@unisa.it (G.A.); 2 Department of Pharmacy, University of Napoli Federico II, Via D. Montesano 49, I-80131 Napoli, Italy; ostacolo@unina.it; 3 Department of Science, University of Basilicata, Viale dell’Ateneo Lucano 10, I-85100 Potenza, Italy; michele.manfra@unibas.it; 4 Food Chemistry, Authentication, Safety and Sensoromic Laboratory, FOCUSS Lab, Mediterranea University of Reggio Calabria, Via Melissari, I-89124 Reggio Calabria, Italy; mariateresa.russo@unirc.it; 5 Dipartimento di Chimica e Tecnologie del Farmaco, Istituto Pasteur-Fondazione Cenci Bolognetti, “Sapienza” Università di Roma, P.le Aldo Moro 5, I-00185 Roma, Italy; antonella.messore@uniroma1.it; 6 Department of Biological, Chemical and Pharmaceutical Sciences and Technologies (STEBICEF), University of Palermo, Via Archirafi 32, 90100 Palermo, Italy; barbara.parrino@unipa.it; 7 European Biomedical Research Institute of Salerno (Ebris), Via De Renzi 50, I-84125 Salerno, Italy

**Keywords:** anthocyanins, LPS, macrophages J774A.1, on-line HPLC-DPPH, oxidative stress

## Abstract

*Citrus* fruits are often employed as ingredients for functional drinks. Among *Citrus*, the variety, “Lempso”, a typical hybrid of the Calabria region (Southern Italy), has been reported to possess superior antioxidant activity when compared to other common *Citrus* varieties. For these reasons, the aim of this study is to investigate in vitro the nutraceutical value of the Tarocco clone, “Lempso”, highlighting its anti-inflammatory and antioxidant potential. A post-column 2,2′-diphenyl-1-picrylhydrazyl (DPPH•) radical scavenging assay for the screening of antioxidant compounds in these complex matrices was developed. Subsequently, polyphenolic extract was tested on a murine macrophage cell line under inflammatory conditions. The extract resulted was able to significantly inhibit nitric oxide (NO) and cytokine release and inducible nitric oxide synthase (iNOS) and cycloxygenase-2 (COX-2) expression. The inhibition of these pro-inflammatory factors was associated to Nuclear factor-kB (NF-kB) inhibition. Our results also indicate an anti-oxidant potential of the extract as evidenced by the inhibition of reactive oxygen species (ROS) release and by the activation of the nuclear factor E2-related factor-2 (Nrf-2) pathway in macrophages. The obtained results highlight the anti-inflammatory and antioxidant potential of Lempso extract and its potential use, as a new ingredient for the formulation of functional beverages with high nutraceutical value, providing health benefits to consumers.

## 1. Introduction

The food and beverage industry is more often interested in natural products, because they represent a rich source of bioactive compounds, bearing health-promoting benefits. The regular intake of fruits or vegetables is a valid strategy to prevent the onset of chronic pathologies. This positive effect is related, at least in part, to the content in bioactive phytochemicals in these food products, such as flavonoids. Flavonoids are a large group of structurally related molecules with a chromane-type skeleton and a phenyl substituent at the C2 or C3 position [1]. They can be referred to as glycosides if they are linked to one or more sugar groups, and as aglycones when no sugar group is present. Among the food sources, *Citrus* contain large amount of flavonoids. *Citrus* belongs to the family, Rutaceae, and is the most important fruit tree crop in the world, with an annual production of approximately 123 million tons in 2010 [2]. Several investigations reported a large number of healthy properties of *Citrus* flavonoids, such as hypolipidemic, hypoglycaemic, anti-inflammatory, and antioxidant properties (Di Donna et al., *Journal of Functional Foods*) [3,4,5,6,7]. Many studies have focused their attention on the ability of flavonoids in reducing oxidative stress and inflammation [8,9,10]. Inflammation is a dynamic biological process, which occurs as result of chemical, physical, immunological, and/or biological stimuli, and it involves an enormous expenditure of metabolic energy, damage, and destruction of host tissues, even with risk of sepsis, multiple organ failure, and death. If the inflammation persists, thus becoming chronic, it contributes to the pathogenesis of various diseases (e.g., type 2 diabetes, cardiovascular disease, cognitive impairment, brain atrophy, and also cancer) [11]. Macrophages are involved in the host defence during the inflammatory and immune response. It is known that, in response to lipopolysaccharide, a component of Gram-negative bacteria cell walls, macrophages produce and release inflammatory mediators, including cytokines; pro-inflammatory enzymes, such as inducible nitric oxide synthase (iNOS) and cycloxygenase-2 (COX-2); and highly reactive species, such as nitric oxide (NO) and reactive oxygen species (ROS). Inflammatory cells release a number of reactive species at the site of inflammation, leading to an exaggerated oxidative stress. On the other hand, a number of reactive oxygen/nitrogen species can initiate the intracellular signaling cascade that initiates and/or further enhances proinflammatory gene expression. Thus, inflammation and oxidative stress are closely related pathophysiological events that are tightly linked with one another. In fact, experimental data show the simultaneous existence of low-grade chronic inflammation and oxidative stress in many chronic diseases, like diabetic complications, cardiovascular and neurodegenerative diseases, alcoholic liver disease, and chronic kidney disease [12]. Similarly to the inflammatory response, despite the oxidative response regulating many physiological responses, if not properly regulated, it can also lead to a number of deleterious effects [13]. Among *Citrus*, the variety, “Lempso”, a typical hybrid of Calabria region, showed superior antioxidant activity in vitro [14] with respect to other common *Citrus* varieties, when challenged by the DPPH test. In this regard, the aim of this work was to investigate its antioxidant and anti-inflammatory potential, in a cellular system, on J774A.1 macrophages and its effect in reducing pro-oxidant and pro-inflammatory mediators.

## 2. Materials and Methods

### 2.1. Sample Collection and Characterization

Tarocco clone “Lempso” (*C. sinensis* (L.) Osbeck) fruits were collected from Vivai Antonino Bertolami mother plants field (LameziaTerme, CZ, Italy). Extraction, purification, and quali-quantitative characterization of Lempso extract were performed as previously described [14], but with slight modification. In order to remove sugars and further purify the methanolic extract, solid phase extraction (SPE) was carried out by employing polymeric reversed phase cartridges (33.3 mg mL^−1^, Phenomenex^®®^). The cartridges were equilibrate with 3 mL of CH_3_OH and activated with 3 mL of H_2_O. After the sample loading, washing and elution phases were carried out with 3 mL of H_2_O + 0.1% *v*/*v* TFA and 6 mL of CH_3_OH + 0.1% *v*/*v* TFA, respectively. The eluted fraction was lyophilized for 24 h (LyoQuest-55, Telstar Technologies, Terrassa, Spain).

### 2.2. On-Line HPLC-DPPH Conditions

**Figure 1:**
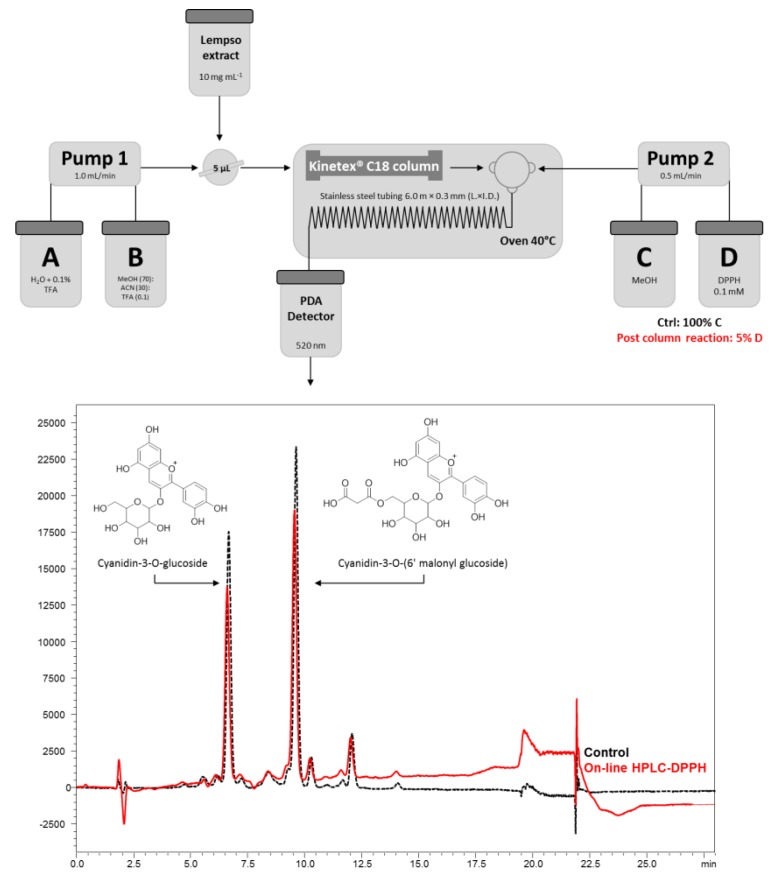
On-line High Performance Liquid Chromatography-Photodiode Array-2,2′-Diphenyl-1-picrylhydrazyl (HPLC-PDA-DPPH) profiles of anthocyanins in Lempso extract. Black and red lines denote the control and post column reaction, respectively.

The separation of anthocyanins was carried out by a Nexera UHPLC system (Shimadzu, Kyoto, Japan) consisting of two LC-30AD pumps, an SPD-M20A photo diode array detector, a CTO-20A column oven, and a SIL-30AC autosampler. UHPLC analysis were performed with a Kinetex^®®^ C18 column (150 × 4.6 mm × 2.6 μm, Phenomenex, Bologna, Italy). The optimal mobile phase consisted of (A) 0.1% TFA/H_2_O *v*/*v* and (B) TFA/MeOH/ACN (0.1/70/30 *v*/*v*) by setting the flow rate at 1.0 mL min^−1^. The injection volume was 5 μL of polyphenolic extract (10.0 mg mL^−1^). Analysis was performed in gradient elution as follows: 0–15.00 min, 10–25% B; 15–17.50 min, 25–40% B; 17.5–18.00 min, 40–95% B; 18–20.00 min, isocratic to 95% B; then, eight minutes for column re-equilibration. Data acquisition was set in the range 190 nm–800 nm and chromatograms were monitored at 520 nm. After the separation, the effluent of the UHPLC column was mixed on-line by a stainless steel Tee union (1/16” in., 0.50 mm bore, Vici-Valco^®®^, Houston, TX 77255, USA) with the DPPH• solution and allowed into a coil reactor (standard stainless steel tubing 1.6 × 0.3 mm × 6.0 m, O.D. × I.D. × L.). Column oven and coil reactor temperatures was set to 40 °C. The radical solution was eluted by an LC-20 AT pump (Shimadzu, Milan, Italy) using as mobile phase (C) CH_3_OH and (D) DPPH• solution and setting the flow rate to 0.5 mL min^−1^ (Figure 1). The DPPH• stock solution was prepared in methanol at a concentration of 0.1 mM, stirred for 10 min at room temperature, and filtered through a 0.45-μm pore PVDF membrane (Millicup-HV, Millipore©, Milan, Italy). The resulting DPPH• reagent was degassed, kept protected from light by wrapping in aluminum foil before use, and changed every 24 h. For the acquisition of a reference, chromatogram (Ctrl) was applied an isocratic elution at 100% C while for the radical scavenger evaluation of single anthocyanins was set 5% of the DPPH• mobile phase (D). The change in anthocyanin peak areas between the control and on-line reaction with DPPH was used to evaluate the antioxidant anthocyanins.

### 2.3. Cells Cultures

#### 2.3.1. J774A.1 Macrophage Cell Line

J774A.1 murine monocyte macrophage cell line (American Type Culture Collection, Rockville, MD, USA) was grown adherent to Petri dishes with Dulbecco’s modified Eagle’s medium (DMEM) supplemented with 10% fetal calf serum (FCS), 25 mM HEPES, 2 mM glutamine, 100 u/mL penicillin, and 100 mg mL^−1^ streptomycin at 37 °C in a 5% CO_2_ atmosphere.

#### 2.3.2. Cell Viability Assay

Cell viability was performed using colorimetric assay based on the 3-(4,5-dimethyl-2-thiazyl)-2,5-diphenyl-2*H*-tetrazolium bromide (MTT) reagent. J774A.1 macrophages (5 × 10^4^ cells/well) were allowed to adhere for 4 h. J774A.1 macrophages were exposed to Lempso flavonoidic extract (LFE) (10–50 μg mL^−1^) for 24 h. Cell viability was then assessed as previously reported using the MTT assay [15]. Briefly, 25 µL of MTT (5 mg mL^−1^) was added and the cells were incubated for 3 h. Thereafter, cells were lysed and the dark blue crystals solubilized with 100 µL of a solution containing 50% (*v*/*v*) *N,N*-dimethylformamide, 20% (*w*/*v*) SDS with an adjusted pH of 4.5. The optical density (OD) of each well was measured with a microplate spectrophotometer (TitertekMultiskan MCC/340) equipped with a 620 nm filter. Cell viability in response to treatment with LFE was calculated as: % dead cells = 100 − (OD treated/OD control) × 100.

#### 2.3.3. Nitrite Determination and Cytofluorimetry Evaluation of iNOS, COX-2, HO-1, and NQO1 Expression in J774A.1 Macrophages

Macrophages J774A.1 were plated in 96 well plate (5.0 × 10^4^/well) and allowed to adhere for 4 h. Thereafter, the medium was replaced with fresh medium alone or containing serial dilutions of LFE (10–50 μg mL^−1^) for 1 h and then simultaneously to lipopolysaccharide from *E.coli* (LPS) (1 μg mL^−1^) for 24 h to detect nitrite (NO_2_^−^), inducible nitric oxide synthase (iNOS), cyclooxygenase-2 (COX-2), heme oxygenase-1 (HO-1), and NAD(P)H quinone dehydrogenase 1 (NQO1) expression. NO (nitric oxide) production in the cell culture supernatants was analyzed as indication of NO_2_^−^ level, by Griess reagent, as previously reported [16,17]. Briefly, 100 µL of cell culture medium were mixed with 100 µL of Griess reagent − equal volumes of 1% (*w*/*v*) sulphanilamide in 5% (*v*/*v*) phosphoric acid and 0.1% (*w*/*v*) naphtylethylenediamine-hydrogen chloride (HCl) and incubated at room temperature for 10 min, and then the absorbance was measured at 550 nm in a microplate reader, Titertek (Dasit, Cornaredo, Milan, Italy). The amount of NO_2_^−^, as μM concentration, in the samples was calculated by a sodium NO_2_^−^ standard curve. iNOS, COX-2, HO-1, and NQO1 expression were evaluated by cytofluorimetry, cells were then collected, washed twice with PBS, and then incubated in Fixing Solution for 20 min at 4 °C and then incubated in Fix Perm Solution for 30 min at 4 °C. Anti-iNOS, anti-COX-2, anti-HO-1, and anti-NQO1 were then added for further 30 min. The secondary antibody was added in Fix solution and cells’ fluorescence was evaluated using a fluorescence-activated cell sorting (FACSscan; Becton Dickinson) and elaborated with Cell Quest software as previously reported [18].

#### 2.3.4. TNF-α and IL-6 Determination in Macrophages J774A.1

Tumor necrosis factor (TNF)-α and interleukin (IL)-6 concentrations in J774A.1 treated with LFE (10–50 μg mL^−1^) and LPS (1 μg mL^−1^) for 18 h, as previously described, were assessed by an Enzyme-Linked Immuno Sorbent Assay (ELISA) assay by using a commercial kit, for murine TNF-α or IL-6, according to manufacturer’s instructions (e-Biosciences, San Diego, CA, USA) as previously reported [19].

#### 2.3.5. Immunofluorescence Analysis with Confocal Microscopy in Macrophages J774A.1

For immunofluorescence assay, J774A.1 cells (3 × 10^5^/well) were seeded on coverslips in a 12 well plate and treated with LFE (25 μg mL^−1^) for 1 h and then simultaneously to LPS (1 μg mL^−1^) for 20 min in order to detect Nuclear Factor-kB (NF-kB) nuclear translocation and for 1 h in order to detect Nuclear Factor (erythroid-derived 2)-like 2 (Nrf2) nuclear translocation. Cells were then fixed with 4% paraformaldehyde in phosphate buffered saline (PBS) for 15 min and permeabilized with 0.1% saponin in PBS for 15 min. After blocking with bovine serum albumin (BSA) and PBS for 1 h, macrophages were incubated with Rabbit anti-phospho p65 antibody (Santa Cruz Biotechnologies, Dallas, TX, USA) or anti-Nrf2 antibody (Santa Cruz Biotechnologies) for 1 h at room temperature. The slides were then washed with PBS for three times and fluorescein-conjugated secondary antibody (FITC) was added for 1 h, 4′,6-diamidine-2′-phenylindole dihydrochloride (DAPI) was used for counterstaining of nuclei. Coverslips were finally mounted in mounting medium and fluorescent images were taken under the Laser Confocal Microscope (Leica TCS SP5, Leica, Wetzalar, Germany) [20].

#### 2.3.6. Measurement of Intracellular ROS

Reactive oxygen species (ROS) formation was evaluated by means of the probe, 2′,7′-dichlorofluorescin-diacetate (H_2_DCF-DA), as previously reported [21]. Briefly, J774A.1 (3.0 × 10^5^/well) macrophages were plated into 24-well plates and treated with LFE (10–50 μg mL^−1^) for 1 h and then simultaneously to LPS (1 μg mL^−1^) for 24 h. J774A.1 macrophages were then collected, washed twice with PBS, and then incubated in PBS containing H_2_DCF-DA (10 µM) at 37 °C. After 45 min, cells’ fluorescence was evaluated using a fluorescence-activated cell sorting (FACSscan; Becton Dickinson) and elaborated with Cell Quest software.

### 2.4. Data Analysis

Data are reported as mean ± standard error mean (s.e.m.) values of at least three independent experiments. Statistical analysis was performed by analysis of variance test, and multiple comparisons were made by Bonferroni’s test. A *p*-value < 0.05 was considered as significant.

## 3. Results

### 3.1. Identification of Main Polyphenols in Lempso Extract and Fast Antioxidant Activity Screening

Several polyphenols were identified in Lempso extract, as can be appreciated from chromatograms extracted at 280 nm for flavonoids and at 520 nm for anthocyanins. The identification was based on UV, accurate MS and MS/MS spectra [22], and a retention time comparison of available standards. The Lempso profile is characterized by methoxy-hydroxicinnamic acids, flavanones, glycosidated flavonols, and anthocyanins (Table S1). Flavanones, Vicenin-2, Narirutin, and Hesperidin, were the most abundant compounds (14.40 ± 0.09, 18.15 ± 0.40, and 179.51 ± 1.54 mg g^−1^, respectively), such as in other *Citrus* varieties [4,5,6], whereas, among anthocyanins, two Cyanidin derivatives, Cyanidin-3-*O*-glucoside and Cyanidin-3-*O*-(6′ malonyl glucoside), were the most abundant anthocyanin molecules present (2.00 ± 0.04 and 2.63 ± 0.05 mg g^−1^, respectively). With the aim to rapidly assess the antioxidant potential of these polyphenolic compounds, we carried out a fast screening approach based on the coupling of chromatographic separation with post-column DPPH radical reaction, in which eluting compounds react with the radical and thus their absorbance (UV/Vis peak area) decreases. Reaction coil length, temperature, reaction time, and DPPH radical concentration were tuned to appreciate the peak areas’ reductions (data not shown). Optimum conditions were found to be: 5 × 10^−6^ M DPPH• reagent; reaction coil of 6 m × 0.3 mm (L. × I.D.) stainless steel tubing; reaction time of 16.96 s; and analysis at 40 °C. Figure 1 highlights the peak area reduction of several compounds. Among different compounds, it can be appreciated that two Cyanidin derivatives were considerably reduced (Cyanidin-3-*O*-glucoside: 24.2 ± 4.2%; Cyanidin-3-*O*-(6′ malonyl glucoside): 22.1 ± 4.3%). This is probably due to the hydroxyl substituents on the B ring, and their high radical scavenging activity. These data are in good accordance with a previous developed method [14].

### 3.2. Evaluation of the Anti-Inflammatory and Antioxidant Potential of LFE in J774A.1 Murine Macrophages

#### 3.2.1. Cells Viability

To elucidate the influence of LFE on J774A.1, cells were treated with LFE (10–50 μg mL^−1^) for 24 h. Our data indicated that J774A.1 macrophages’ viability was not affected by LFE treatment (data not shown).

#### 3.2.2. Lempso Extract Inhibited LPS-Induced NO, iNOS, COX-2, TNF-α, and IL-6 Production

**Figure 2:**
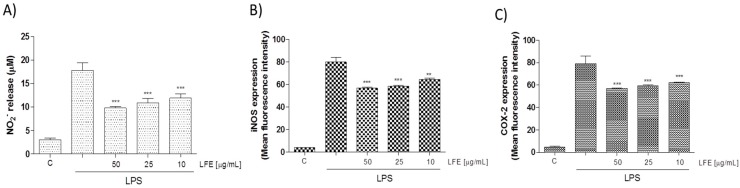
Effect of Lempso flavonoidic extract (LFE) (10–50 μg mL^−1^) on nitric oxide (NO) release, evaluated as NO_2_^−^ (µM), by macrophages J774A.1 stimulated with lipopolysaccharide from *E. coli* (LPS) (Panel A). Effect of LFE (10–50 μg mL^−1^) on LPS-induced inducible nitric oxide synthase (iNOS) (Panel B) and cycloxygenase-2 (COX-2) (Panel C) in macrophages J774A.1. Values are expressed as mean ± s.e.m of at least three experiments, each in triplicate. Comparisons were performed using one-way analysis of variance and multiple comparisons were made by Bonferroni’s test. *** and ** denote *p* < 0.001 and *p* < 0.01 vs. LPS alone.

**Figure 3:**
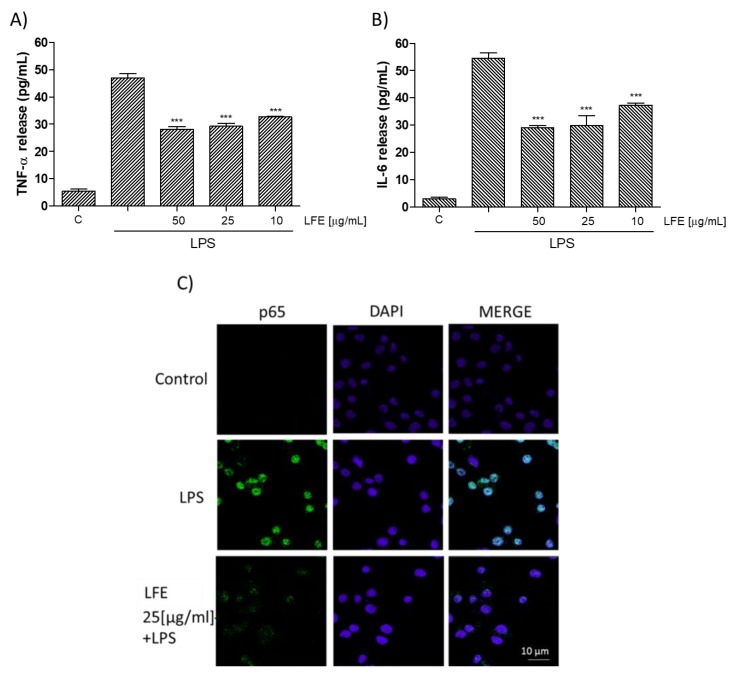
Effect of LFE (10–50 μg mL^−1^) on LPS –induced tumor necrosis factor (TNF)-α (Panel A) and interleukin (IL)-6 (Panel B) production in J774A.1 macrophages. TNF-α and IL-6 production was measured in the supernatants of J774A.1 cells treated with LFE (10–50 μg mL^−1^) and LPS (1 μg mL^−1^) for 18 h by means of Enzyme-Linked Immuno Sorbent Assay (ELISA). Values are expressed as mean ± s.e.m of at least three experiments, each in triplicate. Comparisons were performed using one-way analysis of variance and multiple comparisons were made by Bonferroni’s test. *** denotes *p* < 0.001 vs. LPS alone. Effect of LFE (25 μg mL^−1^) on LPS-induced p65 nuclear translocation (Panel C) in J774A.1 macrophages. Nuclear translocation of Nuclear factor-kB (NF-kB) p65 subunit was detected using immunofluorescence assay at confocal microscopy. Scale bar, 10 µm. Blue and green fluorescence indicate localization of nucleus (DAPI) and p65, respectively. Analysis was performed by confocal laser scanning microscopy.

NO is a signaling molecule, which plays a key role in the pathogenesis of inflammation and is considered as a pro-inflammatory mediator that induces inflammation due to its over production [23]. NO is synthesized from L-arginine in a reaction catalyzed by a family of nitric oxide synthase (NOS) enzymes. Exposure to microbial products, such as LPS or proinflammatory cytokines, induces the expression of the iNOS gene in various inflammatory and tissue cells. Similarly, when LFE (10–50 μg mL^−1^) was added to J774A.1 macrophages 1 h before and simultaneously to LPS stimulation for 24 h, all tested concentrations significantly inhibited NO release in cell medium (*p* < 0.001 vs. LPS; Figure 2A) and induced a decrease also in iNOS expression (*p* < 0.01 vs. LPS; Figure 2B). An interaction between the iNOS and COX pathway represents an important mechanism for the modulation of the inflammatory response [24]. COX-2 is a well-known pro-inflammatory enzyme triggered by agents as LPS, it is involved in the macrophage response and its expression is also influenced by NO [25]. Thus, the effect of LFE on COX-2 expression was evaluated as well. COX-2 protein expression was strongly inhibited at all tested concentrations by LFE (10–50 μg mL^−1^; *p* < 0.001 vs. LPS; Figure 2C). The inflammatory response is characterized also by a massive cytokine production as TNF-α and IL-6 [26]. In our experimental conditions, LFE significantly reduced TNF-α and IL-6 release (*p* < 0.001 vs. LPS; Figure 3A,B respectively) thus further contributing to the anti-inflammatory effect induced by LFE in macrophages.

#### 3.2.3. Lempso Extract Inhibits p65 NF-kB Nuclear Translocation

NF-kB has been considered a prototypical proinflammatory signaling pathway and is involved in the pro-inflammatory mediators evaluated here. NF-κB drives expression of target genes that mediate cell proliferation and release of antimicrobial molecules and cytokines to activate the immune response in macrophages [27]. After p65 phosphorylation, the free NF-κB dimers translocate into the nucleus and bind to specific sequences to regulate the downstream genes’ expression [28]. For this purpose, we labelled p65 with a green fluorescence to track the influence of LFE (at the medium tested concentration of 25 μg mL^−1^) added 1 h before LPS (1 μg mL^−1^) on p65 NF-κB translocation. As shown in Figure 3C, nuclear p65 translocation was increased after 20 min by LPS in J774A.1 treated macrophages, compared to the cellular control (Figure 3C). When LFE (25 μg mL^−1^) was added to macrophages 1 h before and simultaneously with LPS, a decrease of p65 nuclear translocation was observed with respect to LPS alone.

#### 3.2.4. LFE Antioxidant Properties

**Figure 4:**
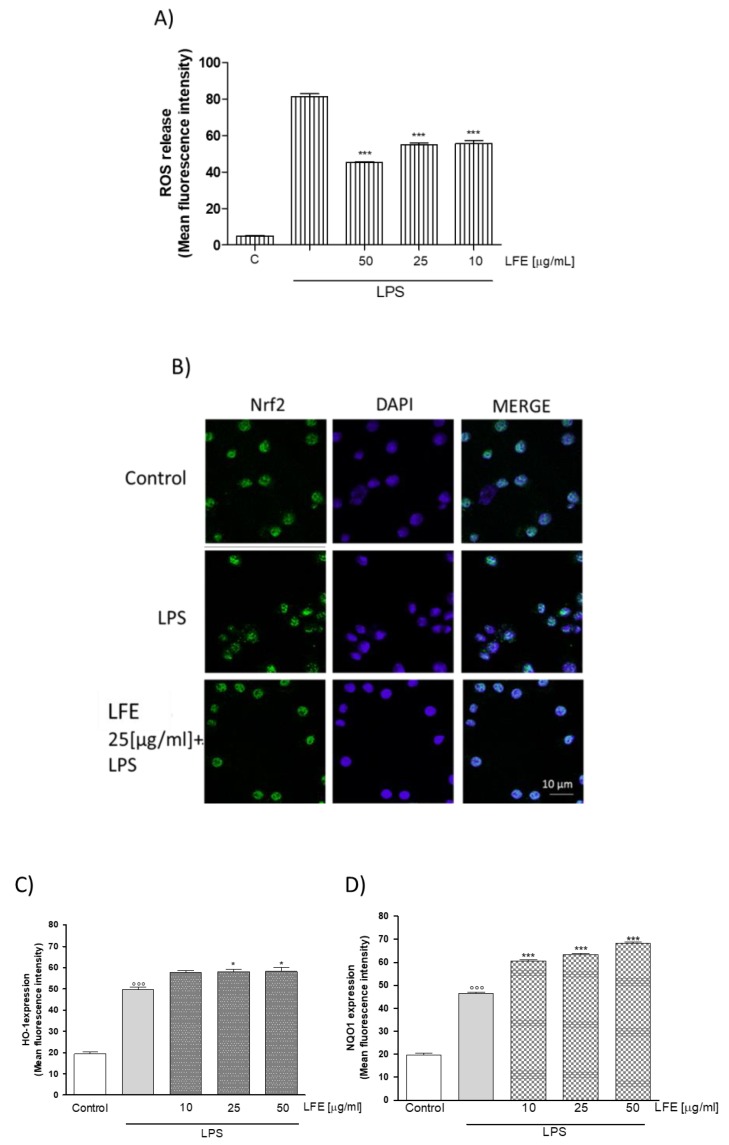
Effect of LFE (10–50 μg mL^−1^) on LPS-induced reactive oxygen species (ROS) in LPS-stimulated J774A.1 macrophages (Panel A). Effect of LFE (25 μg mL^−1^) on NF-E2-related factor-2 (Nrf-2) (Nrf2) nuclear translocation in J774A.1 macrophages (Panel B). Nuclear translocation of Nrf2 was detected using immunofluorescence assay at confocal microscopy. Scale bar, 10 µm. Blue and green fluorescence indicates localization of nucleus (DAPI) and Nrf2, respectively. Analysis was performed by confocal laser scanning microscopy. Effect of LFE (25 μg mL^−1^) on LPS-induced heme oxygenase-1 (HO-1) (Panel C) and on NAD(P)H quinone dehydrogenase 1 (NQO1) (Panel D) in macrophages J774A.1. Values are expressed as mean ± s.e.m of at least three experiments, each in triplicate. Comparisons were performed using one-way analysis of variance and multiple comparisons were made by Bonferroni’s test. °°° denotes *p* < 0.001 vs. control. *** and * denote *p* < 0.001 and *p* < 0.05 vs. LPS alone.

Using the same experimental procedures described above, LFE was challenged for its antioxidant properties in J774A.1 macrophages during inflammation. As expected, and in accordance with the results obtained for the DPPH test, LFE significantly inhibited ROS production in cell medium at all tested concentrations (*p* < 0.001 vs. LPS; Figure 4A). Because oxidative stress is characterized by an imbalance between pro- and anti-oxidant cellular systems, to further elucidate the mechanisms, the effect of LFE was also evaluated on NF-E2-related factor-2 (Nrf-2) pathway activation. Nrf2 is essential for the coordinated transcriptional activation of genes encoding the anti-oxidant enzymes, such as HO-1 and NQO1. Nrf2 was marked with a green fluorescence and LFE, used at a medium concentration (25 μg mL^−1^), reduced Nrf2 nuclear translocation, in comparison with LPS alone, as we can observe from the overlapping of the green fluorescence with the nuclei (marked in blue with DAPI; Figure 4B). Simultaneously, LFE (10–50 μg mL^−1^) increased HO-1 and NQO1 enzyme expression in the selected cell line. In particular, LFE (10–50 μg mL^−1^) significantly increased LPS-induced HO-1 in a concentration range of 50–25 μg mL^−1^ (*p* < 0.05 vs. LPS; Figure 4C) and LPS-induced NQO1 at all tested concentrations (*p* < 0.001 vs. LPS; Figure 4D).

## 4. Discussion

Many of the health benefits of *Citrus* are correlated with the content in flavonoids. In our previous work [14], the hybrid Lempso was compared, in terms of quali-quantitative flavonoid content, to the blood orange variety, “Sanguinello”, and mandarin variety, “Cleopatra”. Lempso possesses a higher amount of flavanones. The Hesperidin amount in particular was higher with than those reported in the literature for other *Citrus* varieties [29,30,31], even though the content of flavanones in *Citrus* can differ based on the harvesting period. From a nutraceutical point of view, this aspect is highly interesting, since Hesperidin and other flavanones possess important antioxidant and anti-inflammatory properties [32,33]. Moreover, anthocyanins are also present in this hybrid. The anti-inflammatory effect of Lempso extract can be partly attributed to the inhibition of NF-κB nuclear translocation. This effect could result from the combined action of the phytocomplex. In fact, as has been shown for the flavanone, Narirutin, in citrus peels [34] and of Cyanidin-type anthocyanins of berries [35], both classes of compounds showed similar effect in RAW 264.7 macrophages. Cyanidin derivatives could also play a role in the inhibition of iNOS and COX-2 expression that are influenced from the inhibition of NF-κB, since iNOS and COX-2 promoters contain NF-κB-binding sites that are required for the maximal response to LPS [36]. On the other hand, flavanones and anthocyanins are able to reduce ROS levels by a double effect. In fact, these compounds, given the large amount of hydroxyl substituents, exert a scavenging activity [37]. Moreover, it has been reported that compounds, such as Cyanidin-3-*O*-Glucoside, activates the Nrf2 pathway, also in stress induced conditions, such as in human endothelial cells being challenged with TNF-α [38]. Thus, the unique profile of Lempsophytocomplex, rich in both flavanones and anthocyanins, is a key factor for its anti-inflammatory and antioxidant activity.

## 5. Conclusions

*Citrus* fruits contain numerous healthy compounds. In this regard, hybridization often leads to the improvement of clones with higher levels of phytochemicals. The Lempso clone has showed a rich content of both flavanones and anthocyanins, which is even higher than other *Citrus* varieties. In vitro assays revealed its anti-inflammatory and antioxidant activity through multiple modalities. These results promote the consumption of Lempso extract as a potential novel functional ingredient for nutraceutical formulations.

## Supplementary Materials

The following are available online at http://www.mdpi.com/2072-6643/10/12/1961/s1, Table S1: Quantitative polyphenol profile of Lempso extract.
