# Supplementary material for: Polyphenolic Extract from Tarocco (Citrus sinensis L. Osbeck) Clone “Lempso” Exerts Anti-Inflammatory and Antioxidant Effects via NF-kB and Nrf-2 Activation in Murine Macrophages
Source: Nutrients. 2018 Dec 11;10(12):1961. doi: 10.3390/nu10121961 (PMC6316643; doi:10.3390/nu10121961)
Supplement: Supplementary file 1 [file nutrients-10-01961-s001.pdf]

Supporting material for the manuscript:

Polyphenolic extract from Tarocco (*Citrus sinensis* L. Osbeck) clone "Lempso" exerts anti-inflammatory and antioxidant effects via NF-kB and Nrf-2 activation in macrophages

Table S1. *Quantitative polyphenol profile of Lempso extract*

Compound	mg g^{-1}
**Methoxy-Hydroxycinnamic Acids and Flavonoids**	
p-Coumaroyl glucose	1.35 ± 0.05
Quercetin 3,4′-di-*O*-β-glucopyranoside	2.06 ± 0.25
Quercetin 7-*O*-glucoside-3-*O*-rutinoside	2.19 ± 0.09
Ferulic acid 4-*O*-glucoside	1.55 ± 0.05
5-8'-Dehydrodiferulic acid	1.59 ± 0.09
p-coumaric (derivate)	1.43 ± 0.07
Apigenin 6,8-di-C-glucoside (Vicenin-2)	14.40 ± 0.09
Naringenin 7- *O* -glucoside (Prunin)	6.40 ± 0.19
Apigenin 7- *O* -apyosil glucoside (Apiin)	2.83 ± 0.15
Quercetin 3- *O* -rutinoside (Rutin)	7.13 ± 0.11
Naringenin 7- *O* -rutinoside (Narirutin)	18.15 ± 0.40
Quercetin-3-[6 ″-(3-hydroxy-3-methylglutaryl)] β-hexoside	0.73 ± 0.06
Isorhamnetin 3-*O*-glucoside 7-*O*-rhamnoside	1.89 ± 0.25
Hesperidin	179.51 ± 1.54
Isosakuranetin 7-rutinoside (Dydimin)	4.61 ± 0.04
**Anthocyanins**	
Cyanidin 3,5-diglucoside	0.17 ± 0.01
Cyanidin 3-sophoroside	0.16 ± 0.01
Delphinidin 3-*O*-glucoside	0.21 ± 0.01
Cyanidin 3-*O*-glucoside	2.00 ± 0.04
Delphinidin-3-*O*-glucoside (Isomer)	0.13 ± 0.01
Peonidin 3-*O*-glucoside	0.26 ± 0.01
Cyanidin-3-*O*-(6' malonyl glucoside)	2.63 ± 0.05

Data are reported as mean ± standard deviation values of at least three independent experiments.
